# Characterization of Suicidal Behaviour with Self-Organizing Maps

**DOI:** 10.1155/2013/136743

**Published:** 2013-06-20

**Authors:** José M. Leiva-Murillo, Jorge López-Castromán, Enrique Baca-García, EURECA Consortium

**Affiliations:** ^1^Department of Signal Theory and Communication, Universidad Carlos III de Madrid, Avenida Universidad 30, 28911 Madrid, Spain; ^2^Department of Psychiatry, Fundación Jiménez Díaz, Avenida Reyes Católicos 2, 28040 Madrid, Spain; ^3^Centre Hospitalier Regional Universitaire de Montpellier, France

## Abstract

The study of the variables involved in suicidal behavior is important from a social, medical, and economical point of view. Given the high number of potential variables of interest, a large population of subjects must be analysed in order to get conclusive results. In this paper, we describe a method based on self-organizing maps (SOMs) for finding the most relevant variables even when their relation to suicidal behavior is strongly nonlinear. We have applied the method to a cohort with more than 8,000 subjects and 600 variables and discovered four groups of variables involved in suicidal behavior. According to the results, there are four main groups of risk factors that characterize the population of suicide attempters: mental disorders, alcoholism, impulsivity, and childhood abuse. The identification of specific subpopulations of suicide attempters is consistent with current medical knowledge and may provide a new avenue of research to improve the management of suicidal cases.

## 1. Introduction

Suicide is one of the leading causes of the Global Burden of Disease, accounting for approximately 1.5% [1]. Every year, about 10 to 20 million people attempt suicide worldwide and one million people actually die because of self-inflicted harm (Data from the WHO, http://www.who.int/mental_health/prevention/suicide). This fact is particularly alarming among the young; suicide causes 6.3% of the global deaths from 10 to 24 years of age [2], and this percentage can rise to 15% in high-income countries. However, suicidal behavior results from a complex interaction between vulnerability factors and environmental events [3], making it difficult to predict or prevent. Many risk and protective factors for suicidal behavior have been consistently identified [4], but predictive models remain imprecise [5]. A better understanding of the hierarchy and organization of variables involved in suicidal behavior may help to improve the detection of potential suicide victims.

Given the complexity of the problem, classical statistical methods are not able to appropriately deal with large sample sizes, high numbers of variables, and strong nonlinear interactions in the data. Modern machine learning and data mining approaches, on the other hand, overcome these limitations and have been successfully applied to computational biology problems such as suicide attempter classification [6]. If sequential data are also available, these methods are able to uncover patterns in the evolution of mental disorders [7].

One of the problems most intensively studied in machine learning is variable selection, which in our particular application consists in identifying the most relevant risk factors in suicidal behavior. Let **x** be a random variable so that a given dataset {**x**_**i**_}_*i*=1,…,*L*_ ∈ *ℛ* ^*D*^ consists of *L* realizations of **x**. Variable selection searches for a subset of variables among the *D* original ones. Feature selection and dimensionality reduction are open problems in machine learning since they are subject to the *no free lunch* principle: a variable selection strategy cannot uniformly outperform other ones in terms of prediction ability [8, 9]. In supervised learning, the variables must be selected according to their ability at predicting an auxiliary variable *y*, which in our case indicates whether the suicidal behavior is observed or not. Variable ranking is a simplification of the variable selection problem that consists in ordering the variables in decreasing order of relevance. 

There is an inherent difficulty regarding feature selection: it is a problem with nonpolynomial complexity (i.e., an NP-problem) unless some particularity of the problem can be exploited. The reason is that all the possible combinations of variables must be evaluated in order to choose the optimal one. For this reason, most of the feature selection methods described in the literature are suboptimal, based on forward selection, backward elimination, or a branch-and-bound approach [10].

An additional difficulty arises when a particular outcome is linked to different subsets of variables depending on the population. This is the case of evaluating the risk of suicide behavior. For this reason, a successful method should be able to (i) cluster the population in a nonlinear way, (ii) use a criterion to find the relevant variables in each subpopulation, and (iii) allow for interacting with the practitioner in the selection of the most clinically relevant information from the different subpopulations.

In this paper, we propose to use a self-organizing map (SOM) to find nonlinear dependencies in the data [11]. Although the SOM is not a state-of-the-art technique in either supervised learning or variable selection, there are a number of reasons that can make it useful in this particular application. First, a SOM provides a simple visualization that facilitates the recognition of the data structure not only to the statistician, but also to the physician. Secondly, it shares some properties with state-of-the-art methods: it can deal with high-dimensional data and nonlinear patterns. Third, although SOMs are intended for unsupervised learning, they have been successfully applied to supervised problems such as bankruptcy prediction [12]. 

The presented work is novel from both the medical and the computational biology points of view. From the medical perspective, the study analyzes a large cohort of 8,699 subjects compiled by five institutions from four different countries that are involved in the EURECA consortium. (The European Research Consortium for Suicide is composed of research groups on suicide from Montpellier, Geneva, Molise, Oviedo, and Madrid.) Each subject has been characterized by 606 variables related to the sociodemographic status of the subjects, as well as their answers to normalized questionnaires that measure hostility, impulsivity, alcoholism, childhood trauma, hopelessness, and so forth. From the computational biology or artificial intelligence point of view, this is the first work, up to the authors' knowledge, that successfully performs a combination of a nonlinear unsupervised learning tool such as the SOM with linear discriminants for variable selection in a supervised learning setting.

In the next section, both the data under study and the method proposed for variable selection are described. In Section 3, we show the results together with the their interpretation from both the machine learning and the medical point of view.  Section 4 closes the paper with the most relevant conclusions. 

## 2. Materials and Methods

 In this section, we first describe the dataset under study. Then, in Section 2.2, the elements from linear discriminants and SOMs that are involved in the study are described.

### 2.1. Dataset

The EURECA consortium have recruited 3,839 suicide attempters and completers over the last years [13]. These clinical teams implemented very similar clinical methods and assessment procedures. Sociodemographic and clinical data of the subjects have been joined in a common database together with the results of assessment tools. All suicide attempters have been hospitalized following an attempt that was defined as follows: “a potentially self-injurious behavior with a nonfatal outcome, for which there is evidence (either explicit or implicit) that the person intended at some (nonzero) level to kill himself/herself.” This definition has been adopted by the National Institute of Mental Health (NIMH) and the main research groups in the UE [14]. Studies were approved by the research ethics committee of each group and conducted according to the tenets of the Declaration of Helsinki. All participants signed an informed consent form after the explanation of the study objective and procedures. In addition to suicide attempters, the dataset includes psychiatric patients with no records of suicide attempts, blood donors, and orthopedic patients with no records of mental disorders or suicidal behaviors and relatives of suicide attempters.

Basic sociodemographic features were collected for all the subjects. Psychiatric diagnoses were assessed using the Diagnostic Interview for Genetics Studies (DIGS) and the Mini International Neuropsychiatric Interview (MINI). Suicide behavior was assessed using the Suicidal Intent Scale (SIS) and the Risk Rescue Rating Scale (RRRS). Suicide ideation was examined with the Scale for Suicidal Ideation (SSI). The following scales validated in different languages were used for the investigation of the intermediate measures of suicidal behavior: the Life History of Aggression (LHA) interview, the Buss-Durkee Hostility Inventory (BDHI), the Spielberger State-Trait Anger Expression Inventory (STAXI), the Barratt Impulsivity Scale (BIS10), the Beck Depression Inventory (BDI), and the Beck Hopelessness Scale (BHS). The Childhood Trauma Questionnaire (CTQ), a retrospective measure of child abuse, and the CAGE questionnaire, for screening of alcohol problems, were also applied. All these variables were included in the dataset. 

### 2.2. Variable Selection and Self-Organizing Maps

A self-organizing map (SOM) is a special kind of unsupervised neural network that consists of a bidimensional grid of units, each one characterized by a vector of the same dimension as the data. The grid can follow either a square or an hexagonal pattern, the latter being the most popular kind [11].

Training the network takes place iteratively using each training data point in two stages. In the first one, a best matching unit (BMU) is found so that its Euclidean distance (other distances have also been used. See [12] for an example with the * information* distance) to the data point is minimal. In the second stage, the centroid together with the BMU neighbors are moved in the direction of the data sample according to the rule

(1) mi(n+1)=mi(n)+α(n)h(w,i)(x(n)−mi(n)),

where **m**_*i*_^(*n*)^ is the value of the *i*th cell vector at instant *n*, *α* ^(*n*)^ is the update rate, and *h*(*w*, *i*) is the neighborhood kernel between the BMU *w* and cell *i*.

One of the main applications of SOMs is clustering, because a SOM consists of a set of centroids as an alternative to the set of centres provided by, for example, *k-means*. This ability for clustering is of interest for this particular application because we aim at identifying homogeneous groups of subjects characterized by different groups of variables and risk factors. Additionally, the two-dimensional layout of the centres allows for further interpretation of the clustering.

In order to rank the variables, according to classical statistics, one of the simplest ways is to use the Fisher discriminant [15]. For a binary decision problem, the Fischer discriminant of a variable *v* is given by

(2) dF(v)=|μ1(v)−μ0(v)|σ1(v)+σ0(v).

Fisher's criterion is a linear discriminant. Hence, it is of limited application for variable selection in problems in which certain variables are strongly related to the auxiliary variable if the relationship is nonlinear. On the other hand, SOMs allow us to find risk factors by considering relationships among variables that can have a nonlinear nature. As mentioned previously, each centroid is updated by the data point that is closest at a given iteration. An auxiliary variable can be * masked* when building the data. However, its value is updated according to (1) and then visualized.

In order to use the SOM structure for variable ranking and selection, we look for the variables in which the distance between the value at the * hottest* unit in the map and the mean value of the negative class is the highest. In other words, we define a new discriminant, similar to Fisher's, given by the expression

(3) dSOM(v)=|c^(v)−μ0(v)|σ1(v)+σ0(v),

where $\hat{c}(v)$ is the value of the aforementioned centroid for variable *v*. Given that the value of the auxiliary variable of a given centroid is built based on the data matching that center, one can expect that the value of the variables in the * hottest* center—in terms of the auxiliary variable—is representative.

## 3. Results and Discussion

In the following, we apply the method described previously to the study of the variables most strongly related to suicidal behaviour. This variable indicates whether the subject has ever attempted suicide. There are 3213 positive and 1983 negative examples; the rest are undefined. We have trained an hexagonal SOM with 16 × 12 cells using the SOM Toolbox for Matlab [16]. A sequential training has been applied, with a linearly decreasing learning factor *α* ^(*n*)^ that achieves convergence after running through the data for 4 times. A Gaussian neighbourhood function *h*(*w*, *i*) has been used to train the SOM. The map is initialized in a deterministic way, setting the unit values to the eigenvectors of the covariance matrix of the data, that is, to the principal components. All the auxiliary variables in the dataset, that is, the ones referring to suicidal behaviour, such as SSI, RRRS, and SIS variables—see Section 2.1—are masked when finding the BMU, although their value is updated according to (1). Missing values are ignored in both the computation of the distances and the application of the update rule.

**Figure 1:**
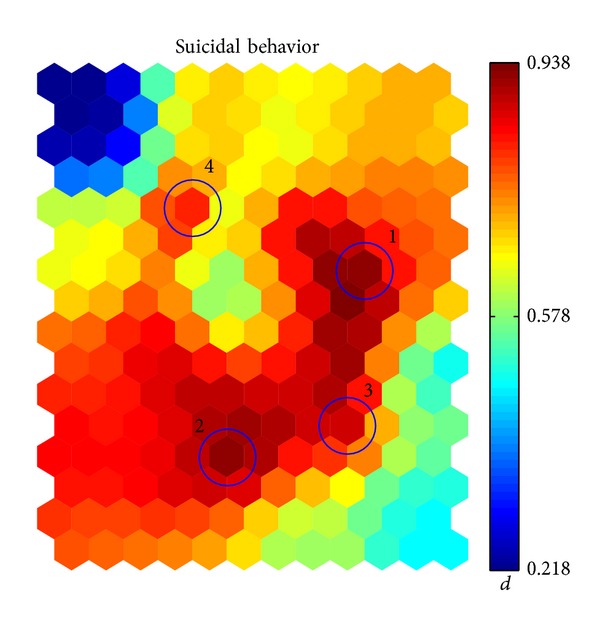
Maps obtained for the * suicidal behavior* auxiliary variable.

Once the map has been trained, we plot in Figure 1 the value of the * suicidal behavior* variable. In the figure, the most outstanding peaks or * hot spots* are highlighted in decreasing order of relevance. The criterion followed to choose these spots is descriptive rather than rigorous. This way, we stress the potential of this tool for building an interactive tool to assist the medical practice, in which the human intuition about the shape of the map is more important than a mathematical criterion for the detection of peaks. In particular, peaks 1, 2, and 3 have been chosen as the vertices of the *L*-shaped wide spot in the map; then, the 4th peak is chosen as the most outstanding isolated spot.

**Figure 2:**
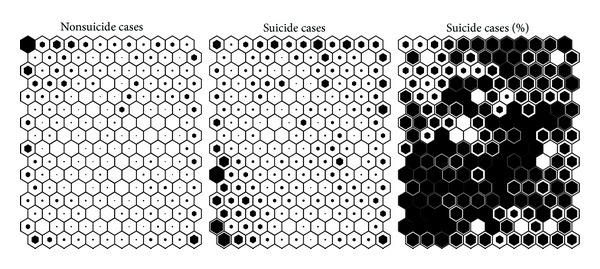
Histograms for the * suicidal behavior* variable. Left: negative samples; center: positive samples; right: percentage of positive samples in each cell.

Once the SOM is trained, each subject can be projected in the map by finding the closest cell in Euclidean distance, so that a histogram of correspondences is built. We show in Figure 2 the histograms of patients with and without a history of suicidal behavior, as well as the histogram with the percentage of subjects with suicide behavior per cell. Each bar in the left and middle 2-D histograms shows the amount of data samples (subjects) that are closer to that cell than to any other. There is a high correspondence between the map with the variable values (Figure 1) and the histogram with the percentages (Figure 2, right).

We have computed the *d*_SOM_ discriminant for the cell with the highest value of the *suicidal behavior* variable, that is, the one with the hottest color in Figure 1. Once the variables with more than 90% missing values are ignored, the list of variables with the highest value is shown in Table 1. The most significant variables are related to mental disorder, which is consistent with the a priori medical knowledge that most mental disorders are associated with greater odds for attempting suicide. In contrast, the Fischer discriminant is unable to find these variables as relevant: these variables have been ranked by Fischer at locations 62, 98, 109, and 80.

**Figure 3:**
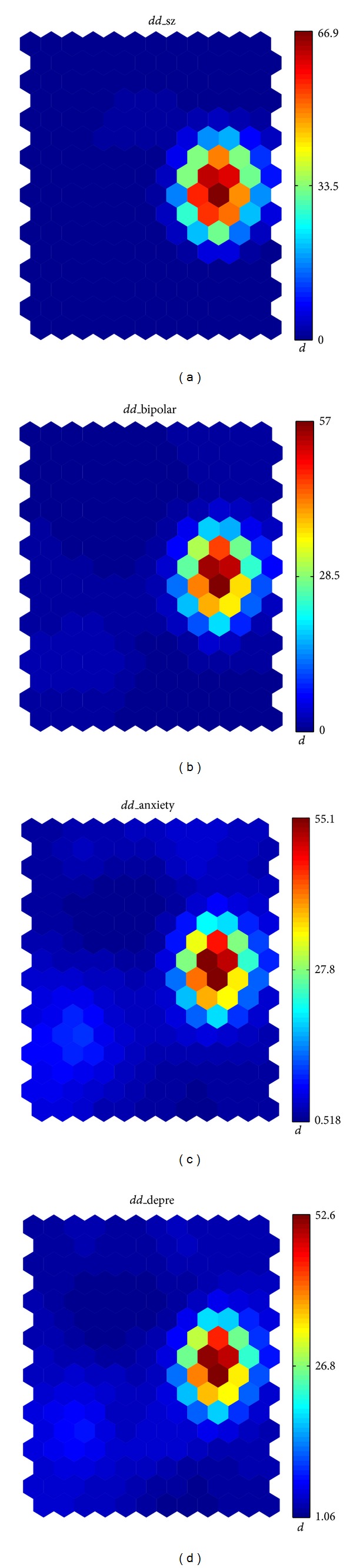
Distribution of the variables with highest *d*_SOM_ value.

We show the map corresponding to these four most relevant variables in Figure 3. The high correlation between these maps and the one shown in Figure 1 is clear.

**Figure 4:**
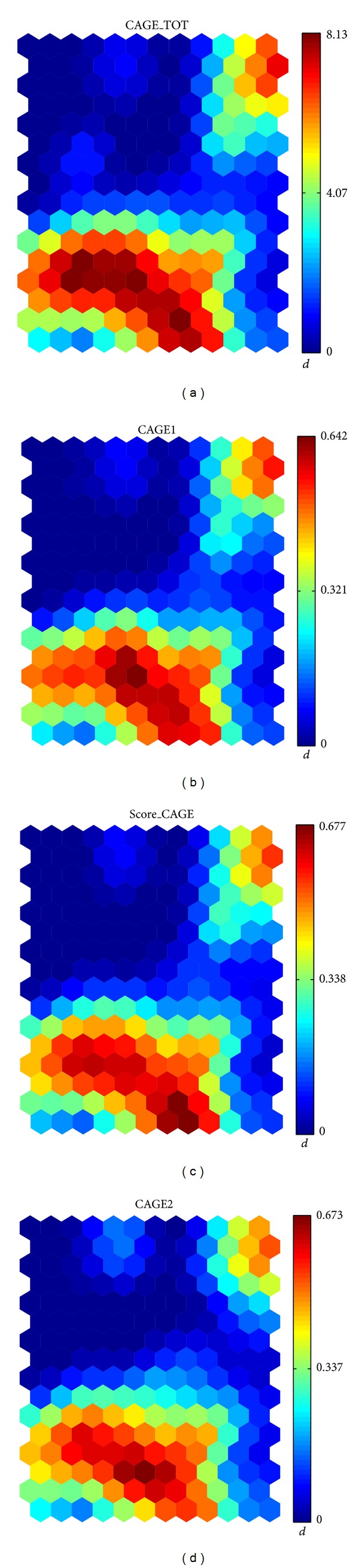
Distribution of the variables with the second highest *d*_SOM_ value in the *suicidal behavior* study.

The second most outstanding cell according to Figure 1 gives the highest value of *d*_SOM_ to variables *CAGE_TOT, CAGE1, Score_CAGE,* and *CAGE2*. The meanings of these variables are given by the answers to the CAGE questionnaire described in Table 2. Variables CAGE1 and CAGE2 are the answers to questions 1 and 2; CAGE_TOT is the summation of the four answers; SCORE_CAGE summarizes the total CAGE score in a yes-no category for alcohol problems. The distributions of these variables' values across the map are shown in Figure 4. The values of the discriminants are shown in Table 3.

**Figure 5:**
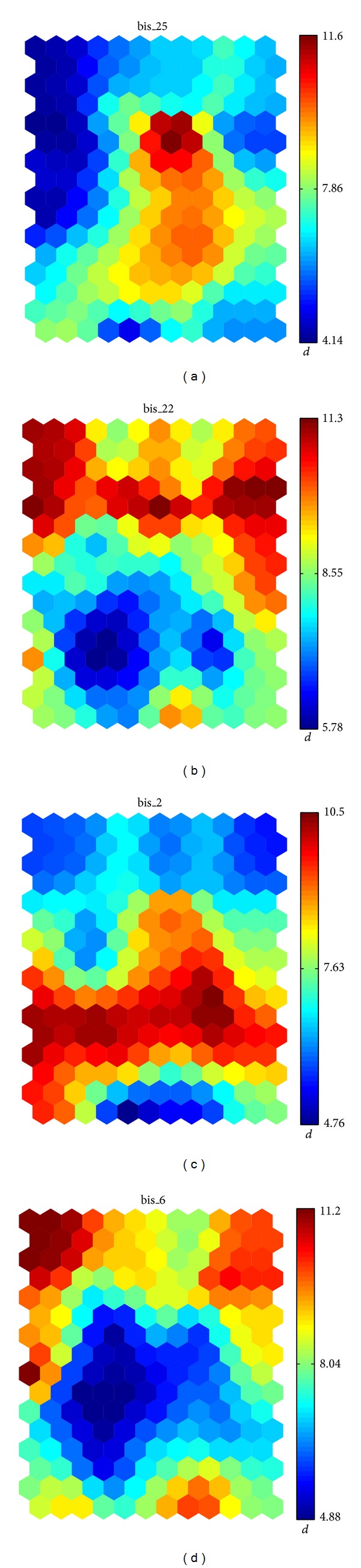
Distribution of the variables with the highest *d*_SOM_ at the third peak of the *suicidal behavior* SOM.

The ranking of the most relevant variables related to the third peak of the map is headed by the questions of the BIS questionnaire described in Table 4. The statistics of the variables are shown in Table 5, and the corresponding maps are displayed in Figure 5. As the figure reveals, the maps corresponding to variables bis_22 and bis_6 show a negative correlation with respect to the map in Figure 1; that is, their values are low in the location of the third peak. This is due to the fact that questions bis_22 and bis_6 are formulated in a negative way; that is, agreement is related to the absence of impulsivity, unlike questions bis_25 and bis_2, in which a positive answer is related to impulsivity. According to (3), the discriminant can also be large by means of a cell value *c*(*v*) lower than the average *μ*_0_(*v*).

**Figure 6:**
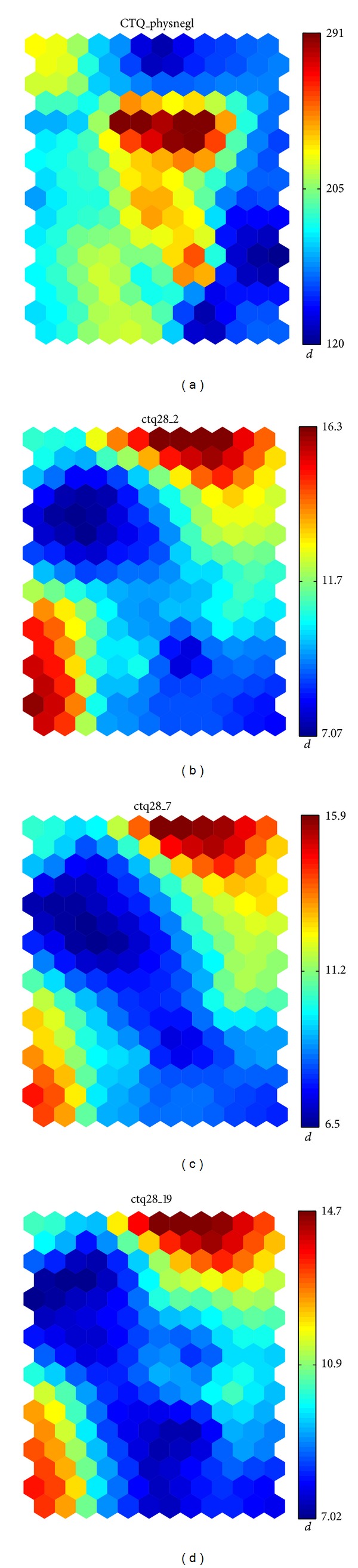
Distribution of the variables with the highest *d*_SOM_ value at the fourth peak.

The exploration of the fourth peak reveals the presence of CTQ questions as the most relevant variables. Their meanings are described in Table 6; the statistics and maps are shown in Table 7 and Figure 6, respectively. Again, we observe a negative correlation in the maps corresponding to variables ctq28_2, ctq28_7, and ctq28_19, due to the fact that those questions are formulated in negative terms (a positive answer is opposite to neglect).

## 4. Conclusions

 We have described a novel technique for variable selection based on self-organizing maps. The technique has been applied to a dataset containing socioeconomical, psychological, and clinical variables from a set of subjects that include suicide attempters. We make use of a discriminative criterion inspired by the Fisher discriminant to extract the variables most strongly related to the suicidal behavior. The map obtained for the *suicidal behavior* variable follows a complex structure with multiple peaks that can be interpreted as the existence of different groups or subpopulations of suicide attempters, with specific features. The study has revealed four groups of factors related to the four peaks observed in the SOM: mental disorders, alcoholism, impulsiveness, and childhood abuse, respectively. These results agree with current knowledge about the risk factors of suicide, which stresses the validity and usefulness of the described approach. The method can be extended to other problems of clinical interest in which heterogeneous risk factors are associated with a particular outcome in the population. The ability of the SOM to visualize the structure of high-dimensional data makes the method suitable for developing tools for the clinical practice, where a deep understanding of the underlying statistics by the practitioner is not needed.

## Figures and Tables

**Table 1:** Variables with the highest value for the *d*_SOM_ discriminant in the strongest peak of the SOM.

Variable	$\hat{c}$	*μ*_0_	*μ*_1_	*σ*_0_	*σ*_1_	*d*_SOM_	*d*_*f*_
Schizophrenia	6.7072	0.1882	1.1908	1.2119	2.9261	1.5754	0.2423
Bipolar	5.8649	0.3417	1.1503	1.2263	2.9354	1.3271	0.1943
Anxiety	5.6541	0.8943	1.7824	1.5440	3.4972	0.9753	0.1762
Depression	5.4166	0.8631	1.8613	1.3602	3.3084	0.9442	0.2138

**Table 2:** Questions of the CAGE test.

	Question
CAGE1	Have you ever felt you needed to cut down on your drinking?
CAGE2	Have people annoyed you by criticizing your drinking?
CAGE3	Have you ever felt guilty about drinking?
CAGE4	Have you ever needed a drink as eye-opener in the morning (⋯)?

**Table 3:** Variables with the highest value for the *d*_SOM_ discriminant at the second most outstanding cell.

Variable	c^	*μ*_0_	*μ*_1_	*σ*_0_	*σ*_1_	*d*_SOM_	*d*_*f*_
CAGE_TOT	2.0134	0.1405	0.7163	0.6342	1.2751	0.9810	0.3016
CAGE1	0.6422	0.0470	0.2175	0.2118	0.4129	0.9526	0.2729
Score_CAGE	0.6141	0.0389	0.2163	0.1936	0.4120	0.9496	0.2928
CAGE2	0.6152	0.0471	0.2234	0.2120	0.4169	0.9034	0.2804

**Table 4:** Questions of the BIS test involved in the third peak of the SOM. The subject rates the sentences according to the scale: (1) rarely/never, (2) occasionally, (3) often, and (4) almost always.

	Question
bis_25	I spend or charge more than I earn
bis_22	I finish what I start
bis_2	I do things without thinking
bis_6	I am self-controlled

**Table 5:** Variables with the highest value for the *d*_SOM_ discriminant at the third most outstanding cell.

Variable	$\hat{c}$	*μ*_0_	*μ*_1_	*σ*_0_	*σ*_1_	*d*_SOM_	*d*_*f*_
bis_25	2.8591	1.3364	1.8414	0.7431	1.1184	0.8180	0.2713
bis_22	1.7817	3.2021	2.6053	0.8455	1.1487	0.7123	0.2993
bis_2	3.0516	1.7894	2.2266	0.8153	1.0474	0.6776	0.2347
bis_6	1.8546	3.1744	2.2745	0.8897	1.0772	0.6710	0.4575

**Table 6:** Variables from the CTQ questionnaire. Possible answers are (1) never, (2) rarely, (3) occasionally, (4) often, and (5) almost always.

	Question
CTQ_physnegl	Physical neglect subscale
CTQ28_2	I knew there was someone to take care of me and protect me
CTQ28_7	I felt loved
CTQ28_19	People in my family felt close to each other

**Table 7:** Variables with the highest value for the *d*_SOM_ discriminant at the fourth most outstanding cell.

Variable	$\hat{c}$	*μ*_0_	*μ*_1_	*σ*_0_	*σ*_1_	*d*_SOM_	*d*_*f*_
CTQ_physnegl	14.1902	8.9753	8.4325	3.7455	3.7974	0.6914	0.0720
CTQ28_2	1.7132	3.6056	3.7707	1.3350	1.4141	0.6884	0.0601
CTQ28_7	1.6115	3.3764	3.3393	1.3357	1.4242	0.6395	0.0135
CTQ28_19	1.7293	3.3107	3.1269	1.3010	1.4036	0.5847	0.0680
